# COVID-19: immunopathogenesis and Immunotherapeutics

**DOI:** 10.1038/s41392-020-00243-2

**Published:** 2020-07-25

**Authors:** Li Yang, Shasha Liu, Jinyan Liu, Zhixin Zhang, Xiaochun Wan, Bo Huang, Youhai Chen, Yi Zhang

**Affiliations:** 1 Biotherapy Center, The First Affiliated Hospital of Zhengzhou University, 450052 Zhengzhou, China; 2 Institute of Health Management, Health Management Center, Sichuan Provincial People’s Hospital, University of Electronic Science and Technology of China, 611731 Chengdu, China; 3 Shenzhen Laboratory of Human Antibody Engineering, Institute of Biomedicine and Biotechnology, Shenzhen Institutes of Advanced Technology, Chinese Academy of Sciences, 518055 Shenzhen, China; 4 Department of Immunology & National Key Laboratory of Medical Molecular Biology, Institute of Basic Medical Sciences, Chinese Academy of Medical Sciences (CAMS) & Peking Union Medical College, 100005 Beijing, China; 5 Department of Pathology and Laboratory Medicine, School of Medicine, University of Pennsylvania, Philadelphia, PA 19104 USA

**Keywords:** Infectious diseases, Immunotherapy

## Abstract

The recent novel coronavirus disease (COVID-19) outbreak, caused by severe acute respiratory syndrome coronavirus 2 (SARS-CoV-2), is seeing a rapid increase in infected patients worldwide. The host immune response to SARS-CoV-2 appears to play a critical role in disease pathogenesis and clinical manifestations. SARS-CoV-2 not only activates antiviral immune responses, but can also cause uncontrolled inflammatory responses characterized by marked pro-inflammatory cytokine release in patients with severe COVID-19, leading to lymphopenia, lymphocyte dysfunction, and granulocyte and monocyte abnormalities. These SARS-CoV-2-induced immune abnormalities may lead to infections by microorganisms, septic shock, and severe multiple organ dysfunction. Therefore, mechanisms underlying immune abnormalities in patients with COVID-19 must be elucidated to guide clinical management of the disease. Moreover, rational management of the immune responses to SARS-CoV-2, which includes enhancing anti-viral immunity while inhibiting systemic inflammation, may be key to successful treatment. In this review, we discuss the immunopathology of COVID-19, its potential mechanisms, and clinical implications to aid the development of new therapeutic strategies against COVID-19.

## Introduction

The current coronavirus disease 2019 (COVID-19) outbreak is a worldwide emergency, as its rapid spread and high mortality rate has caused severe disruptions. The number of people infected with severe acute respiratory syndrome coronavirus 2 (SARS-CoV-2), the causative agent of COVID-19, is rapidly increasing worldwide. Patients with COVID-19 can develop pneumonia,^1,2^ severe symptoms of acute respiratory distress syndrome (ARDS), and multiple organ failure.^2–4^

Increasing evidence shows that immune patterns are closely associated with disease progression of patients infected with viruses. A decrease in peripheral T cell subsets is a unique characteristic in patients with severe acute respiratory syndrome (SARS).^5^ In recovered patients, a rapid restoration of peripheral T cell subsets is detected; thus, peripheral T cell number can serve as an accurate diagnostic tool for SARS.^5^ A similar phenomenon was also reported in another study, where the immune system was found impaired during SARS.^6^ In another study, natural killer (NK) cell number was found decreased in patients with Ebola compared with healthy donors.^7^ Proinflammatory cytokines were elevated after Ebola virus disease symptom onset, whereas recovered patients showed low cytokine levels.^8^

With the unraveling of the relationship between immune responses and COVID-19, immune characteristics are now being recognized as potential biomarkers for disease progression as well as potential therapeutic targets for COVID-19. In this review, we summarize the immune characteristics of COVID-19 and discuss the potential mechanisms of SARS-CoV-2-induced immune changes, their effect on disease outcomes, and their implications for potential COVID-19 treatments.

## The immunopathology of COVID-19

**Fig. 1:**
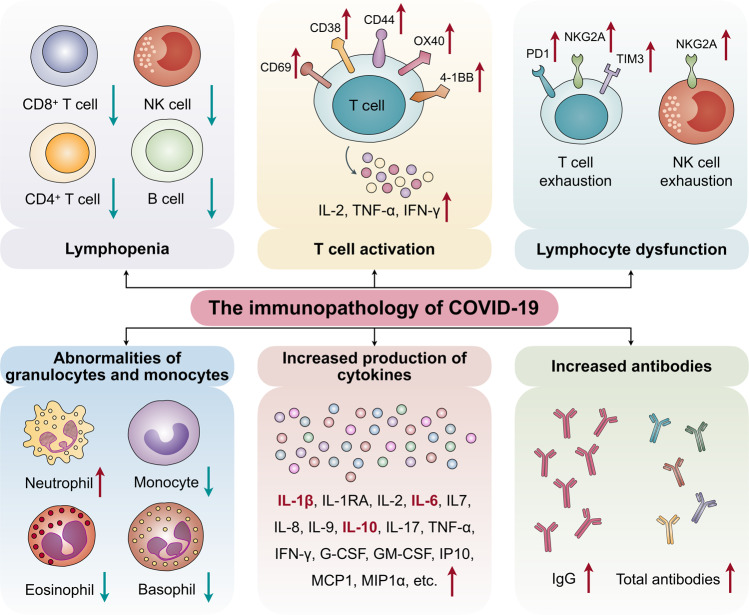
The immunopathology of COVID-19. The immune patterns of COVID-19 include lymphopenia, lymphocyte activation and dysfunction, abnormalities of granulocytes and monocytes, increased production of cytokines, and increased antibodies. Lymphopenia is a key feature of patients with COVID-19, especially in severe cases. CD69, CD38, and CD44 are highly expressed on CD4^+^ and CD8^+^ T cells of patients, and virus-specific T cells from severe cases exhibit a central memory phenotype with high levels of IFN-γ, TNF-α, and IL-2. However, lymphocytes show an exhaustion phenotype with programmed cell death protein-1 (PD1), T cell immunoglobulin domain and mucin domain-3 (TIM3), and killer cell lectin-like receptor subfamily C member 1 (NKG2A) upregulation. Neutrophil levels are significantly higher in severe patients, while the percentage of eosinophils, basophils, and monocytes are reduced. Increased cytokine production, especially of IL-1β, IL-6, and IL-10, is another key characteristic of severe COVID-19. IgG levels are also increased and there is a higher titer of total antibodies

It has been shown that SARS-CoV-2 disrupts normal immune responses, leading to an impaired immune system and uncontrolled inflammatory responses in severe and critical patients with COVID-19. These patients exhibit lymphopenia, lymphocyte activation and dysfunction, granulocyte and monocyte abnormalities, high cytokine levels, and an increase in immunoglobulin G (IgG) and total antibodies. The immune patterns of COVID-19 are outlined in detail in the following sections (Fig. 1).

### Lymphopenia

Lymphopenia is a key feature of patients with COVID-19, especially in severe cases. Patients with severe COVID-19 are more likely to exhibit lymphopenia on admission, indicating a significant predictor for severe patients.^9,10^ Patients also show a marked reduction in CD4^+^ T, CD8^+^ T, NK, and B cell number.^2,11–13^ Lymphocyte percentages were found to be lower than 20% in severe cases.^14^ Further analysis showed a significant decrease in T cell counts, especially CD8^+^ T cells in severe cases compared with mild cases.^15^ Qin et al.^12^ reported that the percentage of memory helper T cells (CD3^+^CD4^+^CD45RO^+^) is also decreased in severe cases compared with non-severe cases. These data indicate that lymphopenia can be used as an indicator of disease severity and prognosis of patients with COVID-19. Nevertheless, lymphopenia was present in some non-severe and pregnant cases;^2,16–24^ however, the percentage of non-severe patients with lymphopenia is significantly lower than that of severe patients.^25,26^ Interestingly, the B cell number is within the normal range,^12^ which is similar to our study findings,^27^ indicating that impaired B cells are not as significant as impaired T or NK cells.

### Lymphocyte activation and dysfunction

T cell activation was investigated in some COVID-19 cases.^28^ In one study with 128 convalescent samples, the CD8^+^ T cell response occurred more frequently than the CD4^+^ T cell response. Furthermore, virus-specific T cells from severe cases presented with a central memory phenotype and high levels of interferon (IFN)-γ, tumor necrosis factor (TNF)-α, and interleukin (IL)-2 compared with that of the mild group.^29^ Zhou et al.^30^ reported that CD69, CD38, and CD44 are highly expressed on CD4^+^ and CD8^+^ T cells of patients with COVID-19 compared with healthy controls. Moreover, the expression of OX40 and 4-1BB, key molecules for promoting clonal expansion^31^ and priming immune responses,^32^ is remarkably increased, especially in severe patients, indicating that T cells are likely to be activated in patients with COVID-19. Another study also demonstrated that activated CD4^+^ and CD8^+^ T cells are present in the blood before the relief of symptoms.^33^

In addition, T cells in patients with COVID-19 show exhaustion phenotypes. programmed cell death protein-1 and T cell immunoglobulin domain and mucin domain-3 levels on CD8^+^ T cells are increased in overtly symptomatic stages compared with the prodromal stage, and peak levels are detected in severe conditions.^26^ Moreover, killer cell lectin-like receptor subfamily C member 1 receptor expression on cytotoxic lymphocytes, including NK and CD8^+^ T cells, is elevated.^34,35^ Thus, elevated exhaustion levels and reduced functional diversity of T cells may predict severe progression in patients with COVID-19.^36^

### Abnormalities of granulocytes and monocytes

The number of granulocytes and monocytes is also abnormal in patients with COVID-19. Neutrophils and the neutrophil-to-lymphocyte ratio—usually important indicators for severe cases and poor clinical outcome^37^—are significantly higher in severe patients than in non-severe patients.^9,10,12,15^ In another study, 38% of 99 cases from Wuhan were found to have increased neutrophil levels.^22^ Meanwhile, a reduced percentage of eosinophils, basophils, and monocytes was observed in severe patients.^12,37^

### Increased production of cytokines

Increased cytokine production is another key characteristic of severe COVID-19. Most severe COVID-19 cases exhibit an extreme increase in inflammatory cytokines, including IL-1β, IL-2, IL-6, IL-7, IL-8, IL-10, granulocyte-colony stimulating factor (G-CSF), granulocyte macrophage-colony stimulating factor (GM-CSF), interferon-inducible protein-10 (IP10), monocyte chemotactic protein 1 (MCP1), macrophage inflammation protein-1α, IFN-γ, and TNF-α,^2,3,12,15^ representing a “cytokine storm”. In particular, IL-1β, IL-6, and IL-10 are the three most elevated cytokines in severe cases.^25,26^ Our study also showed that cytokine levels, including IL2, IL-4, IL-6, IL-10, TNF-α and IFN-γ, are elevated in severe and critical COVID-19 cases, particularly IL-6 and IL-10, which showed a dramatic increase in levels.^27^

In non-severe patients, cytokine levels, including IL-1β, IL-1RA, IL-2R, IL-6, IL-7, IL-8, IL-9, IL-10, IFN-γ, TNF-α, G-CSF, GM-CSF, IP10, MCP1, are also upregulated in the blood^2,12,22^ but are significantly lower than those in severe patients.

### Increased antibodies

The detection of SARS-CoV-2-specific antibodies (IgM and IgG) combined with nucleic acid assays provides the basis of COVID-19 diagnosis. Interestingly, Zhang et al.^37^ found that an increased IgG response is closely associated with disease severity, indicating a simple complementary marker to discriminate between severe and non-severe cases. Another study also showed that <40% of patients have the presence of antibodies in the first 7 days of illness, which then rapidly increases to 100% by day 15 after onset. A higher titer of total antibodies was independently associated with a worse clinical outcome of patients with COVID-19, and was significantly quicker than that of IgM and IgG.^38^ In another study, within 19 days after symptom onset, 100% of patients tested positive for antiviral IgG.^39^ Nicol et al.^40^ reported that the sensitivity for IgG detection, >14 days after onset of symptoms, was 100%, and the specificity was also excellent for IgG; however, the specificity was significantly different between IgA (78.9%) and IgM (95.8%). These observations indicate that B cell activation and proliferation in patients with COVID-19, especially in severe cases, is correlated with poor outcome, which is similar to our study data showing that patients with relatively high B cell levels have poor survival.^26,27^

### Comparison with SARS-CoV-induced immunopathology

Immune responses to SARS-CoV infection are initiated by the innate immune system, which recognizes pathogens and induces proinflammatory cytokines to trigger the immune response, and is followed by responses of the adaptive immune system, consisting of T cells that can directly kill virus-infected cells and B cells that produce pathogen-specific antibodies. Cytokines are produced during the immune response, which further attracts proinflammatory cells, such as macrophages and neutrophils, to the sites of infection to induce an inflammatory response. Although these responses are crucial to virus clearance, they can also damage normal host tissues.^41^ Nevertheless, there is no solid evidence showing that changes including lymphopenia, lymphocyte dysfunction, and granulocyte and monocyte abnormalities are present in SARS, indicating that they are specific immune profiles of COVID-19.

## Potential mechanisms of SARS-CoV-2-induced immunopathology

**Fig. 2:**
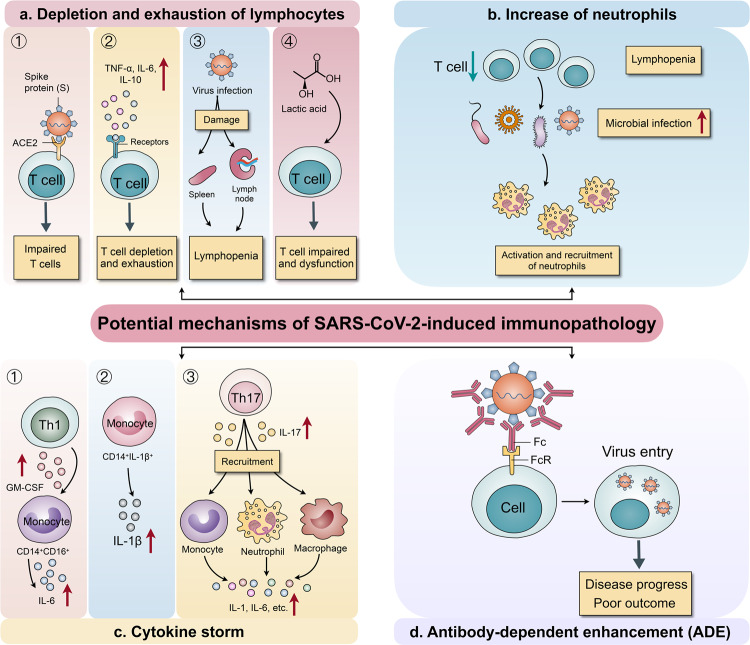
Potential mechanisms of SARS-CoV-2-induced immunopathology. **a** The potential mechanisms of depletion and exhaustion of lymphocytes. (1) ACE2 receptor expression on lymphocytes, especially on T cells, promotes SARS-CoV-2 entry into lymphocytes. (2) A concomitant increase in inflammatory cytokine levels promotes the depletion and exhaustion of T cells. (3) SARS-CoV-2 directly damages lymphatic organs, including the spleen and lymph nodes, inducing lymphopenia. (4) Increased lactic acid levels inhibit the proliferation and dysfunction of lymphocytes. **b** Lymphopenia may lead to infection with microbe, further promoting the activation and recruitment of neutrophils in the blood. **c** The potential mechanisms of cytokine storm induction. (1) CD4^+^ T cells can be rapidly activated into Th1 cells that secret GM-CSF, further inducing CD14^+^CD16^+^ monocytes with high IL-6 levels. (2) An increase in the CD14^+^IL-1β^+^ monocyte subpopulation promotes increased IL-1β production. (3) Th17 cells produce IL-17 to further recruit monocytes, macrophages, and neutrophils and stimulate other cytokine cascades, such as IL-1β and IL-6 among others. **d** A neutralizing monoclonal antibody targeting the virus can enhance virus entry into cells via the Fc region of the antibody bound to the Fc receptor (FcR) on cells; this is correlated with disease progression and poor outcomes of patients with COVID-19

Elucidating the mechanisms underlying immune changes in patients with COVID-19 is important for guiding therapeutic strategies. The potential mechanisms of SARS-CoV-2-induced immune changes are discussed below (Fig. 2).

### Depletion and exhaustion of lymphocytes

There are several potential mechanisms responsible for lymphocyte depletion and dysfunction. (1) It has been reported that SARS-CoV-2 infects human respiratory epithelial cells through interactions between S proteins (Spike glycoprotein) on the virus with angiotensin-converting enzyme 2 (ACE2) receptors.^33^ Furthermore, SARS-CoV-2 can directly infect T cells and macrophages, which is a key feature of SARS-CoV-mediated pathogenesis.^41^ Thus, we hypothesize that ACE2 receptor expression on lymphocytes, especially on T cells, promotes SARS-CoV-2 entry into lymphocytes. (2) Another study showed that the decreased T cell number is inversely correlated with TNFα, IL-6, and IL-10 levels, indicating that an increase in inflammatory cytokine levels can promote the depletion and exhaustion of T cell populations concomitant with disease progression.^42^ (3) Moreover, the SARS-CoV-2 virus may directly destroy lymphatic organs including the spleen and lymph node, as spleen atrophy and lymph node necrosis were observed, further inducing lymphopenia.^14,43,44^ (4) Finally, an increased level of lactic acid was detected in the blood of patients with severe COVID-19,^9^ which may inhibit lymphocyte proliferation.^14^

### Increased neutrophils

Regarding neutrophil upregulation in patients with COVID-19, we can theorize a close association with lymphopenia. It is known that infection with microbe can directly induce neutrophil recruitment to tissue sites.^45,46^ Therefore, the impaired lymphocytes in patients with COVID-19 may easily lead to an infection with microbe, further promoting the activation and recruitment of neutrophils in the blood of patients.

### Cytokine storm

A dramatic increase in cytokines levels over a short time period can cause a cytokine storm, which has been regularly encountered in cancers treated with chimeric antigen receptor T (CAR-T) cells.^47,48^ In patients with severe COVID-19, there is an abundance of cytokine production, inducing a cytokine storm in addition to a series of adverse reactions in the human body. Thus, understanding the mechanisms underlying cytokine storms is necessary. Upon infection with SARS-CoV-2, CD4^+^ T cells can be rapidly activated into pathogenic T helper (Th) 1 cells that secret GM-CSF, which further induces CD14^+^CD16^+^ monocytes with high IL-6 levels and accelerates inflammation.^30^ Single cell analysis revealed that immune cell interaction is characterized by an increase in a subpopulation of CD14^+^IL-1β^+^ monocytes in patients with COVID-19, which may promote increased IL-1β production.^49^ Increasing evidence has shown that Th17 cells producing inflammatory cytokine IL-17 further recruit monocytes/macrophages and neutrophils to the site of infection and stimulate other cytokine cascades, such as IL-1β and IL-6 among others.^50^ Moreover, the Th17 response was detected and confirmed in patients with COVID-19.^11^ Previous studies showed that monocytes/macrophages can produce inflammatory cytokines during murine hepatitis virus strain-3 infection.^51,52^ However, whether SARS-CoV-2 triggers a cytokine storm via monocytes/macrophages requires more detailed studies.^26^ In addition, eosinophils play a direct role in fighting RNA viruses, and can release a large amount of cytokines. Among these cytokines, IL-6 is a key mediator for the development of cytokine storm in COVID-19 cases.^53^

### Antibody-dependent enhancement

The Antibody-dependent enhancement (ADE) of virus infection is a phenomenon in which preexisting sub-neutralizing antibodies enhance virus entry and replication, and has been observed for various viruses, including the Ebola and Dengue viruses.^54,55^ A neutralizing monoclonal antibody targeting the receptor-binding domain of the S protein of Middle East respiratory syndrome (MERS) virus has been shown to enhance virus entry into cells via the Fc portion of the antibody bound to the Fc receptor (FcR) on cells.^43^ This supports the relationship between antibody upregulation and poor outcome of patients with COVID-19. Nevertheless, the ADE-mediated inflammatory response and association of pre-existing antibodies with disease progression and severity in COVID-19 require further investigation.

## Clinical implications of SARS-CoV-2-induced immunopathology

**Fig. 3:**
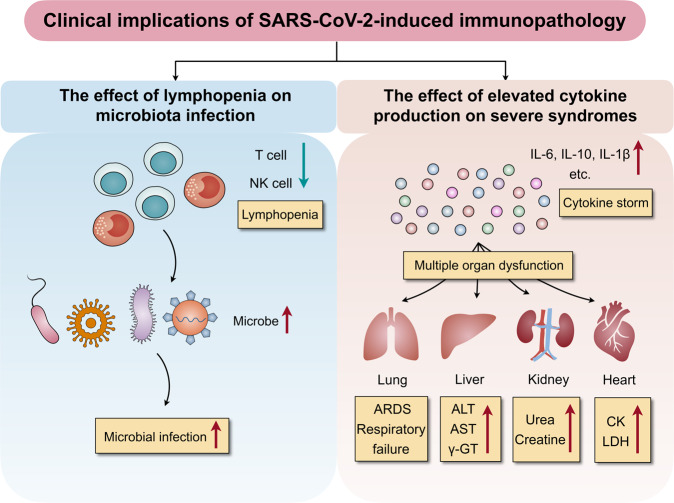
Clinical implications of SARS-CoV-2-induced immunopathology. Patients with COVID-19 and presenting with lymphopenia are more prone to infections with the microbe, which leads to disease progression and increased severity. In addition, cytokine storms can initiate inflammatory-induced multiple organ dysfunction, including lung injury that can lead to ARDS, respiratory failure, liver injury with alanine aminotransferase (ALT), aspartate aminotransferase (AST), and γ-glutamine transferase (γ-GT) upregulation, kidney injury with increased urea and creatine levels, and heart injury with increased creatine kinase (CK) and lactate dehydrogenase (LDH) levels

The SARS-CoV-2-mediated immune signatures may influence the outcomes of patients with COVID-19 in the clinic. In the following sections, we outline the correlation between immune changes and clinical outcomes of COVID-19 (Fig. 3).

### The effect of lymphopenia on microbial infection

Lymphopenia is a common feature in patients with COVID-19 and may be a critical factor associated with disease severity and mortality.^21^ There is a crosstalk between immune homeostasis and microbe in several diseases.^56^ (1,3)-β-d-glucan, a well-known polysaccharide, is a key structural component of the fungal cell wall. In our previous study, we found that in patients with severe COVID-19 and low lymphocyte levels, (1,3)-β-d-glucan levels are significantly higher than in patients with high lymphocyte levels.^27^ Moreover, most patients infected with microbe had low lymphocyte levels, indicating that patients with lymphopenia are more prone to microbial infection.^27^ Chen et al.^3^ demonstrated that multiple microbes could be cultured from one patient, which is similar to our study findings. Overall, the findings indicate that microbial infection in patients with COVID-19 promotes disease progression and severity.

### The effect of elevated cytokine production on clinical manifestations

Increasing evidence shows that viral infection can induce severe syndromes of shock and organ failure;^8,57^ this phenomenon was also investigated for COVID-19. Chen et al.^3^ reported that 43 (43%) patients presented with abnormal liver function and alanine aminotransferase and/or aspartate aminotransferase levels above the normal range; most of the 99 patients had abnormal myocardial zymograms showing elevated creatine kinase and lactate dehydrogenase levels; and some had renal function damage with elevated urea nitrogen or creatinine levels. Other studies also showed that some patients had multiple organ dysfunction, which may have resulted from SARS-CoV-2-mediated immune responses.^9,11,18,58^ A cytokine storm can initiate viral sepsis and inflammatory-induced lung injury, leading to ARDS, respiratory failure, shock, organ failure, and potentially death.^59^ Moreover, in severe COVID-19 cases, high levels of pro-inflammatory cytokines may lead to shock, tissue damage, or multiple organ failure.^42^ Continuous high levels of cytokines (CXCL10, CCL7, and IL-1RA) are associated with lung dysfunction and injury as well as fatal outcomes.^60^ The rise in the serum levels of pro-inflammatory cytokines are also observed in SARS-CoV and MERS-CoV infection, suggesting a potentially similar cytokine storm-mediated disease severity.^61,62^ In our study, IL-6 levels were found closely associated with biomarkers of liver, kidney, and heart dysfunction.^27^ We also found that with an increase in IL-10, biomarkers for γ-glutamine transferase levels are upregulated. Taken together, the above data suggest that severe syndromes of multiple organ dysfunction are closely correlated with elevated cytokine levels, which could be utilized as a promising therapeutic or prevention target for patients with COVID-19 and severe syndromes.

## Potential immunotherapeutic strategies for COVID-19

As discussed above, there is strong evidence supporting a close association between SARS-CoV-2-induced immunopathology and poor survival of patients with COVID-19. Unfortunately, antivirals, glucocorticoids, and immunoglobulin treatments have not shown a significant improvement on the survival of patients with severe COVID-19.^9^ Therefore, targeting the specific COVID-19 immune profiles, such as by enhancing lymphocytes or inhibiting inflammation, are promising treatment strategies for severe cases. Enhancing lymphocytes includes NK cell-based therapy, immunomodulators, or convalescent plasma therapy (Table 1). To inhibit inflammation, strategies such as mesenchymal stem cell (MSC)-based therapy, regulatory T (Treg) cell-based therapy, blood purification, blockade of IL-6 signaling, and Janus kinase (JAK) inhibitors can be employed (Table 1).

**Table 1:** Potential therapeutic strategies for COVID-19

Action	Therapeutic strategy	Agent	Function	Comment
Enhancing lymphocytes	NK cell-based therapy	NK cells	Anti-viral defense, enhances immune response	Kleo Pharmaceuticals Inc. develops NK cell combination therapy
	Immunomodulators	IFN alfa-2a, 2b	Stimulates innate antiviral responses	Clinical trial has been initiated (ChiCTR2000029387)
		*Pseudomonas aeruginosa*		NA
		Thymosin		NA
	Convalescent plasma therapy	Convalescent plasma	Antibodies from convalescent plasma inhibit viremia	Patients treated with convalescent plasma show decreased mortality rate
Inhibiting inflammation	MSC-based therapy	MSCs	Anti-inflammatory effect, repairs pulmonary epithelial cell damage	Clinical trial is ongoing
	Treg cell-based therapy	Tregs	Anti-inflammation	Cellenkos Inc. develops a novel allogenic cell therapy (CK0802)
	Blood purification	Blood purification	Prevention of cytokine storm	Approved for severe and critical cases by China
	Blockade of IL-6 signaling	Tocilizumab	Targets IL-6R signaling to relieve inflammation	ChiCTR2000029765
		Sarilumab	Blocks anti-human IL-6R	Phase 2/3 trial evaluating the safety and efficacy of sarilumab for COVID-19 treatment is underway
	Anti-inflammatory agents	Baricitinib	Inhibits JAK signaling to decrease cytokine levels	Combined with direct-acting antivirals reduces viral replication and aberrant host inflammatory response
		Fedratinib		NA
		Ruxolitinib		NA
		DHODH inhibitors	Inhibits virus replication, attenuates cytokine storm	Effective in infected animals

*COVID-19* Coronavirus disease 2019, *NK* natural killer, *IFN* Interferon, *NA* not applicable, *MSC* mesenchymal stem cell, *Treg* regulatory T, *IL-6* Interleukin-6, *IL-6R* Interleukin-6 receptor, *JAK* Janus kinase, *DHODH* Dihydroorotate dehydrogenase

### NK cell-based therapy

Accumulating evidence as well as clinical studies have demonstrated the promising anti-tumor effects of NK cell-based immunotherapy,^63^ where NK cells activate an antigen-independent immune response against cancer cells. For COVID-19 treatment, NK cell-based therapy has been approved and employed in China to contribute to anti-viral defense and enhance the immune response. Kleo Pharmaceuticals Inc. has entered into a research collaboration with Green Cross LabCell to develop NK cell combination therapy and rapidly advance testing of an advanced technology platform as a potential therapy for patients with COVID-19 (http://www.kleopharmaceuticals.com).

### Immunomodulators

Immunomodulators are substances that affect immune system function, representing a potential therapeutic strategy for COVID-19. For instance, pegylated IFN alfa-2a and 2b, approved for the treatment of hepatitis B and C virus, can be used to stimulate innate antiviral responses in patients infected with SARS-CoV-2. Clinical trials involving interferons have already been initiated for testing the approved anti-hepatitis C virus combination therapy of a pegylated interferon plus ribavirin for patients with COVID-19 (ChiCTR2000029387).^64^ Other immunomodulators, such as *Pseudomonas aeruginosa* and thymosin, may be effective for COVID-19 treatment due to their immune regulatory functions.

### Convalescent plasma therapy

Convalescent plasma from patients that have recovered from a viral infection can be used as therapy for patients with COVID-19 without severe adverse events. One possible explanation for the efficacy of convalescent plasma therapy is that the antibodies present in convalescent plasma may inhibit viremia.^65^ Indeed, several studies have shown lower mortality rates for patients treated with convalescent plasma than those who did not receive this therapy.^66–68^ However, Cheng et al. demonstrated that it is more effective to administer convalescent plasma during early stages of the disease.^66^ However, despite potentially rapid availability, the deployment of convalescent plasma will be limited because transfusions are typically performed in hospital settings and may require large infusion volumes. In addition, there are adverse events for plasma transfusions, including mild fever, allergic reactions, life-threatening bronchospasm, transfusion-related acute lung injury, and circulatory overload in patients with cardiorespiratory disorders, which must be carefully tracked.^69^

### MSC-based therapy

Transplantation of MSCs may represent another effective method for alleviating SARS-CoV-2-related immunopathology and treating SARS-CoV-2-induced pneumonia.^68^ MSCs possess limitless self-renewal and multipotency, with anti-inflammatory effects that defend against cytokine storm, repair pulmonary epithelial cell damage, and promote alveolar fluid clearance.^43,70^ Preclinical and clinical studies on MSC-based therapy have confirmed its safety and efficacy. Several clinical trials testing MSC-based therapy for patients with severe COVID-19 have been recently approved and initiated in China, and thus more clinical data will be available in the future.^43^ Chen et al.^70^ reported that MSCs significantly improve the survival rate of patients with H7N9-induced ARDS. Given that H7N9 and COVID-19 present with similar complications (e.g., ARDS, lung failure, and multiple organ dysfunction), MSC-based therapy has potential as a treatment strategy against COVID-19.

### Treg cell-based therapy

The dysregulated inflammatory processes caused by SARS-CoV-2 in patients with severe COVID-19 are partially due to the dysfunction of Tregs, which are responsible for inhibiting inflammation. Considering that CD4^+^ T cell immunotherapy is a promising approach for treating CD8^+^ T cell dysfunction in chronic infections and cancer,^71^ adoptive therapy with Tregs may be an effective strategy for COVID-19 treatment by balancing inflammation in the lung tissue. Cellenkos Inc. has developed novel allogenic cell therapy (CK0802) consisting of Tregs for overcoming immune dysfunction by resolving chronic inflammation. In addition, this Treg cell expresses a homing marker for lung tissue to interrupt the SARS-CoV-2-induced cytokine storm. In a preclinical study using a lung injury model, the effects of Treg transplantation included decreased inflammatory T cells, inflammatory cytokines IL-17 and IL-6, and alveolar hemorrhage as well as regeneration of lung epithelium and alveoli (http:www.cellenkosinc.com).

### Blood purification

Blood purification is a treatment that aims to remove toxins and wastes from the body to treat diseases. Currently, the Chinese government has approved blood purification for treating severe and critical patients with COVID-19 due to its effective prevention of cytokine storms and suppression of inflammation (http://www.nhc.gov.cn/yzygj/new_index.shtml).

### Blockade of IL-6 signaling

IL-6 is an important factor in inducing cytokine storm, as it drives the overactive inflammatory response. Thus, targeting the IL-6/IL-6 receptor (IL-6R) signaling pathway is a promising strategy for relieving inflammation symptoms.^72^ Tocilizumab, a humanized anti-IL-6 receptor antibody, has been developed and approved for the treatment of rheumatoid arthritis and juvenile idiopathic arthritis.^73,74^ Moreover, tocilizumab has been shown to be effective against cytokine release syndrome resulting from CAR-T cell infusion against B cell acute lymphoblastic leukemia.^75^ Blocking anti-human IL-6R by administering tocilizumab has been approved by China for COVID-19 treatment; a clinical trial (ChiCTR2000029765) demonstrated that fever is rapidly controlled and respiratory function is improved in patients with severe COVID-19 and that all patients recovered.^5,43^ Xu et al.^76^ reported that there are no obvious adverse reactions and that 19 patients (90.5%) were discharged on average 13.5 days after tocilizumab treatment, with the rest recovering well.

Sarilumab is a fully-humanized monoclonal antibody that inhibits the IL-6 signaling pathway by binding to and blocking IL-6R. A phase 2/3, randomized, double-blind, placebo-controlled trial is currently underway to evaluate the safety and efficacy of sarilumab in in adult patients with serious COVID-19 complications (https://www.drugs.com/clinical_trials/first-patient-outside-u-s-treated-global-kevzara-sarilumab-clinical-trial-program-patients-severe-18499.html).

### Anti-inflammatory agents

Inhibitors of the JAK signaling pathway are powerful anti-inflammatory agents that are likely to be effective against the consequences of elevated cytokine levels in various diseases. Baricitinib, fedratinib, and ruxolitinib are three selective JAK inhibitors that have been approved for application in rheumatoid arthritis and myelofibrosis. Moreover, baricitinib combined with direct-acting antivirals (lopinavir or ritonavir and remdesivir) were employed for patients with COVID-19 and showed reduced viral replication and aberrant host inflammatory response.^50,77^ In addition, dihydroorotate dehydrogenase (DHODH) inhibitors were shown to be effective in infected animals not only by inhibiting viral replication but also by alleviating cytokine storms, indicating that they can potentially be used for treating patients with COVID-19.^78^ Nevertheless, the potential risks and benefits of cytokine inhibition must be carefully addressed to guide the continuation or halting of such treatments.^79^

## Conclusions

Patients with COVID-19 exhibit lymphopenia and high cytokine levels, which can be considered potential biomarkers for disease progression. The specific immune profiles of COVID-19 can further induce microbial infection and multiple organ dysfunction. Therefore, improving lymphopenia and reducing inflammation may represent effective therapeutic strategies for patients with COVID-19.
